# Supplementary material for: Exploring Clinical Correlates of Metacognition in Bipolar Disorders Using Moderation Analyses: The Role of Antipsychotics
Source: J Clin Med. 2021 Sep 24;10(19):4349. doi: 10.3390/jcm10194349 (PMC8509459; doi:10.3390/jcm10194349)
Supplement: Supplementary file 1 [file jcm-10-04349-s001.zip › Supplementary Figure S6_revPR.pdf]

Supplementary Figure S6. Distribution of objective cognition according to the level of cognitive complaints in the observed dataset

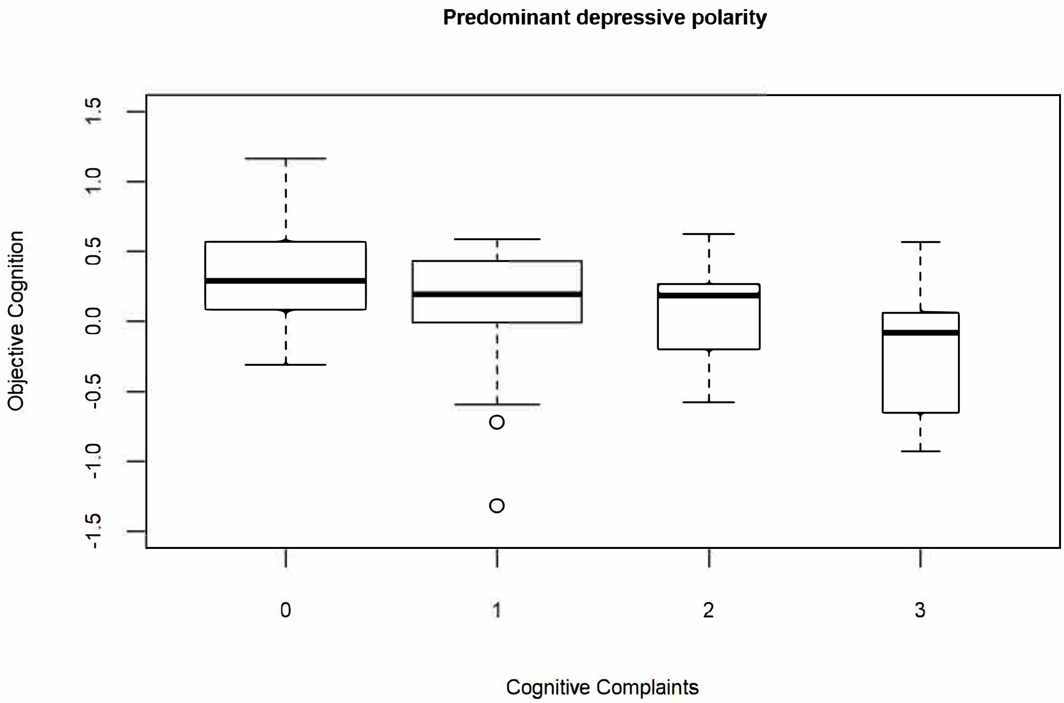

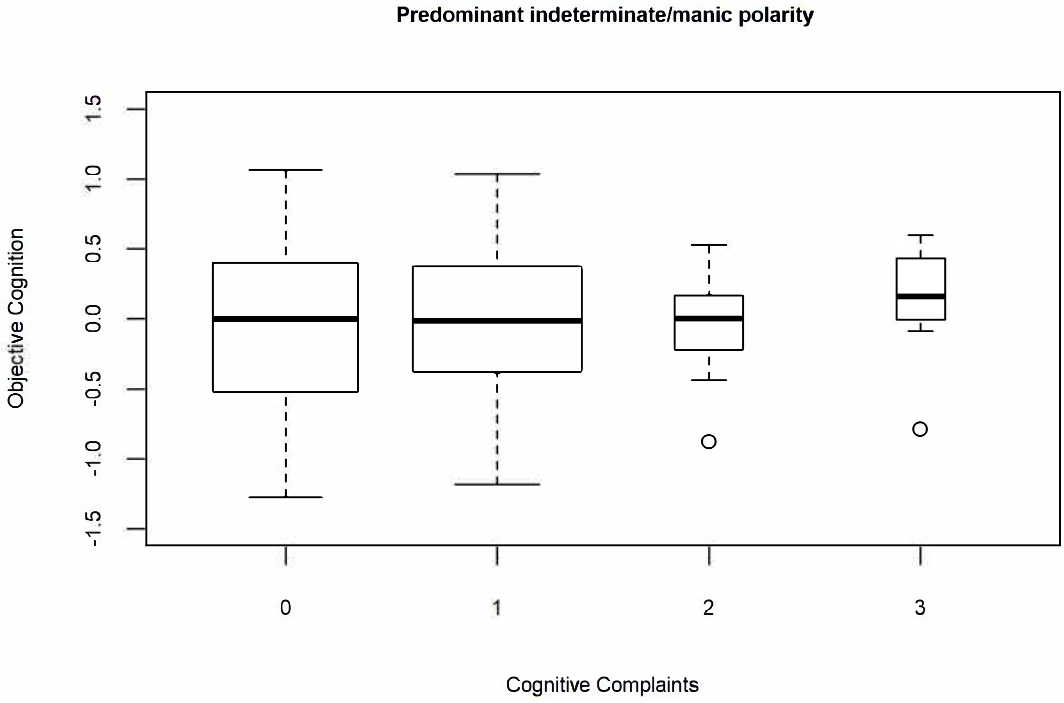

The width of the box is proportional to the sample size in each level of cognitive complaints
