# Supplementary material for: Exploring Clinical Correlates of Metacognition in Bipolar Disorders Using Moderation Analyses: The Role of Antipsychotics
Source: J Clin Med. 2021 Sep 24;10(19):4349. doi: 10.3390/jcm10194349 (PMC8509459; doi:10.3390/jcm10194349)
Supplement: Supplementary file 1 [file jcm-10-04349-s001.zip › Supplementary Information S1_revPR.pdf]

Supplementary Information S1. Test-retest reliability and internal consistency for the clinical scales

CGI-S has good test-retest reliability (intraclass correlation coefficient of 0.98) [1]. Its internal consistency cannot be computed as it is a single-item evaluation. YMRS has substantial test-retest reliability (kappa of 0.64 & 0.75) [2], and good internal consistency (Cronbach alpha of 0.74) [3]. MADRS has moderate to substantial test-retest reliability (kappa of 0.47 & 0.65) [2], and excellent internal consistency (Cronbach alpha of 0.98) [4]. STAI-Y-A (state subscale) has excellent test-retest reliability (Pearson coefficient of 0.96) [5] and good internal consistency (Cronbach alpha from 0.868 to 0.921) [6]. BIS-10 has good internal consistency (Cronbach's alpha of 0.72) [7]. Test-retest reliability is not known for this version of the scale, but it is good for other versions (Pearson product moment correlation coefficients above 0.80) [8]. CTQ has excellent internal consistency (Cronbach alpha of 0.97) [9] and test-retest reliability (intraclass correlation coefficient of 0.81 & 0.91) [10]. FAST has excellent internal consistency (Cronbach alpha of 0.91) and test-retest reliability (intra-class correlation coefficient of 0.98) [11]. MARS has good internal consistency (Cronbach alpha of 0.75) [12] and moderate test-retest reliability over a 12 months period (Pearson correlation coefficients of 0.52) [13].

References for Supplementary Information S1

1. Spearing, M.K.; Post, R.M.; Leverich, G.S.; Brandt, D.; Nolen, W. Modification of the Clinical Global Impressions (CGI) Scale for Use in Bipolar Illness (BP): The CGI-BP. Psychiatry Res. 1997, 73, 159–171, doi:10.1016/s0165-1781(97)00123-6.

2. Sajatovic, M.; Gaur, R.; Tatsuoka, C.; De Santi, S.; Lee, N.; Laredo, J.; Tripathi, S. Rater Training for a Multi-Site, International Clinical Trial: What Mood Symptoms May Be Most Difficult to Rate? Psychopharmacol. Bull. 2011, 44, 5–14.

3. Mühlbacher, M.; Egger, C.; Kaplan, P.; Simhandl, C.; Grunze, H.; Geretsegger, C.; Whit-worth, A.; Stuppäck, C. [Reliability and concordance validity of a German version of the Young Mania Rating Scale (YMRS-D)]. Neuropsychiatr. Klin. Diagn. Ther. Rehabil. Organ Ges. Oster-reichischer Nervenarzte Psychiater 2011, 25, 16–25.

4. Cano, J.F.; Gomez Restrepo, C.; Rondón, M. [Validation of the Montgomery-Åsberg Depression Rating Scale (MADRS) in Colombia]. Rev. Colomb. Psiquiatr. 2016, 45, 146–155, doi:10.1016/j.rcp.2015.08.006.

5. Fountoulakis, K.N.; Papadopoulou, M.; Kleanthous, S.; Papadopoulou, A.; Bizeli, V.; Nimatoudis, I.; Iacovides, A.; Kaprinis, G.S. Reliability and Psychometric Properties of the Greek Translation of the State-Trait Anxiety Inventory Form Y: Preliminary Data. Ann. Gen. Psychiatry 2006, 5, 2, doi:10.1186/1744-859X-5-2.

6. Han, Y.; Fan, J.; Wang, X.; Xia, J.; Liu, X.; Zhou, H.; Zhang, Y.; Zhu, X. Factor Structure and Gender Invariance of Chinese Version State-Trait Anxiety Inventory (Form Y) in University Students. Front. Psychol. 2020, 11, 2228, doi:10.3389/fpsyg.2020.02228.

7. Foullu, S.; Blanc-Foullu, S.; Danet, F.; Dumas, P.; Brunelin, J.; Travart, M.; Elchardus, J.-M.; Saoud, M.; d'Amato, T. [Validation of the French translation of the impulsive nonconformity scale]. L'Encephale 2008, 34, 563–569, doi:10.1016/j.encep.2007.08.010.

8. Juneja, R.; Chaiwong, W.; Siripool, P.; Mahapol, K.; Wiriya, T.; Shannon, J.S.; Petchkrua, W.; Kunanusont, C.; Marriott, L.K. Thai Adaptation and Reliability of Three Versions of the Barratt Impulsiveness Scale (BIS 11, BIS-15, and BIS-Brief). Psychiatry Res. 2019, 272, 744–755, doi:10.1016/j.psychres.2018.12.173.

9. Bernstein, D.P.; Ahluvalia, T.; Pogge, D.; Handelsman, L. Validity of the Childhood Trauma Questionnaire in an Adolescent Psychiatric Population. J. Am. Acad. Child Adolesc. Psychiatry 1997, 36, 340–348, doi:10.1097/00004583-199703000-00012.

10. Simpson, S.; Phillips, L.; Baksheev, G.; Garner, B.; Markulev, C.; Phassouliotis, C.; Alva-rez-Jimenez, M.; McGorry, P.; Bendall, S. Stability of Retrospective Self-Reports of Childhood Trauma in First-Episode Psychosis. Early Interv. Psychiatry 2019, 13, 908–913, doi:10.1111/eip.12700.

11. Rosa, A.R.; Sanchez-Moreno, J.; Martinez-Aran, A.; Salamero, M.; Torrent, C.; Reinares, M.; Comes, M.; Colom, F.; Van Riel, W.; Ayuso-Mateos, J.L.; et al. Validity and Reliability of the Functioning Assessment Short Test (FAST) in Bipolar Disorder. Clin. Pract. Epidemiol. Ment. Health CP EMH 2007, 3, 5, doi:10.1186/1745-0179-3-5.

12. Thompson, K.; Kulkarni, J.; Sergejew, A.A. Reliability and Validity of a New Medication Adherence Rating Scale (MARS) for the Psychoses. Schizophr. Res. 2000, 42, 241–247, doi:10.1016/s0920-9964(99)00130-9.

13. Fialko, L.; Garety, P.A.; Kuipers, E.; Dunn, G.; Bebbington, P.E.; Fowler, D.; Freeman, D. A Large-Scale Validation Study of the Medication Adherence Rating Scale (MARS). Schizophr. Res. 2008, 100, 53–59, doi:10.1016/j.schres.2007.10.029.
