# Supplementary material for: Exploring Clinical Correlates of Metacognition in Bipolar Disorders Using Moderation Analyses: The Role of Antipsychotics
Source: J Clin Med. 2021 Sep 24;10(19):4349. doi: 10.3390/jcm10194349 (PMC8509459; doi:10.3390/jcm10194349)
Supplement: Supplementary file 1 [file jcm-10-04349-s001.zip › Supplementary Information S3_revPR.pdf]

Supplementary Information S3. Diagnostic for imputation of missing data

The percentage of missingness (the number of missing cells for a variable divided by the total number of cells) for the different variables explored in this study is reported in the table below.

Variable	Percentage of missingness
Total number of mood episodes	1.36
Predominant Polarity	1.36
History of psychosis	0.6
MARS[1]	0.33
Objective cognition	0.29
Rapid cycling	0.22
Age at onset	0.12
Any lifetime substance use disorder	0.1
CTQ[2]	0.05
Cognitive complaints	0.05
CGI[3] Severity	0.02
BIS[4]	0.02
FAST[5]	0.01
AGE	0
SEX	0
Educational level	0
Diagnosis	0
MADRS[6]	0
YMRS[7]	0
Antidepressant	0
Anticonvulsant	0
Lithium Carbonate	0
Antipsychotic	0
Anxiolytic	0
Type of WAIS[8]	0

[1]Medication Adherence Rating Scale
[2]Childhood Trauma Questionnaire
[3]Clinical Global Impression scale
[4]Barratt Impulsiveness Scale
[5]Functioning Assessment Short Test
[6]Montgomery Åsberg Depression Rating Scale
[7]Young Mania Rating Scale
[8]Wechsler Adult Intelligence Scale

In order to test for systematic relationships between domains of missing data and participant demographics, we run successive tests with the proportion of missing data for one variable as the dependent variable and one participant demographic characteristic as the independent variable (chi^2-test for sex and Welch's t-tests for age and education). Results are reported in the table below.

Variable	Age		Sex		Education	
Total number of mood episodes / Predominant Polarity	**t(232.6) = 2.8**	***p*=0.005**	chi^2(1) = 0.1	*p* = 0.71	t(232.1) = −0.3	*p* = 0.772
Age at onset	t(9.7) = 1.3	*p* = 0.234	chi^2(1) = 0	*p* = 0.863	t(9.6) = −1.9	*p* = 0.093
History of psychosis	t(68) = 0.2	*p* = 0.826	chi^2(1) = 0.8	*p* = 0.382	t(66.5) = 0.9	*p* = 0.348
Rapid cycling	t(20.1) = 1.1	*p* = 0.289	chi^2(1) = 0.3	*p* = 0.58	t(21.5) = 1.4	*p* = 0.185
CGI[1]	**t(278) = −3.3**	***p* = 0.001**	chi^2(1) = 0	*p* = 1	t(1) = 0.8	*p* = 0.565
Any lifetime substance use disorder	t(7.4) = −0.9	*p* = 0.4	chi^2(1) = 0	*p* = 1	t(7.5) = 0.6	*p* = 0.575

Variable	Age		Sex		Education	
BIS[2]	t(1)=-1.9	p = 0.297	chi²(1)=0	p = 1	t(1) = −1.3	p = 0.414
CTQ[3]	t(3.1)=0.4	p = 0.746	chi²(1)=0	p = 0.843	t(3.4) = −0.8	p = 0.455

[1]Clinical Global Impression scale

[2]Barratt Impulsiveness Scale

[3]Childhood Trauma Questionnaire

The proportion of missing data was associated with sex for no variable. It was also the case for education, in which the proportion of missing data was associated with no variable. The proportion of missing data was associated with age for two variables: participants with missing data for the total number of mood episodes / predominant polarity were older than participants without missing data; participants with missing data for the CGI were younger than participants without missing data. As the associations between age and missingness for the total number of mood episodes /predominant polarity and CGI were in the opposite direction, we conclude a lack of a systematic relationship between domains of missing data and participant demographics.
