# Supplementary material for: Exploring Clinical Correlates of Metacognition in Bipolar Disorders Using Moderation Analyses: The Role of Antipsychotics
Source: J Clin Med. 2021 Sep 24;10(19):4349. doi: 10.3390/jcm10194349 (PMC8509459; doi:10.3390/jcm10194349)
Supplement: Supplementary file 1 [file jcm-10-04349-s001.zip › Supplementary Table S4_revPR.pdf]

Supplementary Table S4. Results for the trivariable ordinal logistic regressions with cognitive complaints as the dependent variable and objective cognition, several successive clinical moderators, and the interaction between objective cognition and the clinical moderator as independent variables. This table reports only the main effect of clinical moderators (the main effect of objective cognition and the interaction between objective cognition and the clinical moderator are reported in Supplementary Table S5 & Table 3 respectively).

**Variable**	**OR (95% CI)**	**Statistic**	**p**	**λ**	**fmi**
Age	0.99 (0.97–1.01)	t(270.1) = −1.1	0.284	0.01	0.017
Sex	0.85 (0.55–1.32)	t(270.6) = −0.7	0.479	0.009	0.016
Educational level	0.92 (0.84–1)	t(270.2) = −1.9	0.063	0.01	0.017
**Diagnosis (Type 2/NOS vs Type 1)**	1.75 (1.11–2.76)	**t(269.6) = 2.4**	**0.017**	**0.012**	**0.019**
Total number of mood episodes	0.98 (0.94–1.03)	t(69.8) = −0.8	0.446	0.55	0.563
Predominant Polarity (Indeterminate/Manic vs Depressive)	0.73 (0.42–1.25)	t(141.5) = −1.1	0.252	0.3	0.31
Age at onset	0.99 (0.96–1.02)	t(262) = −0.8	0.447	0.034	0.042
**History of psychosis**	0.56 (0.31–0.99)	**t(177.1) = −2**	**0.047**	**0.218**	**0.226**
Rapid cycling	1.18 (0.49–2.84)	t(229.3) = 0.4	0.719	0.11	0.118
**CGI Severity**	1.84 (1.38–2.44)	**t(267.9) = 4.2**	**<0.001**	**0.017**	**0.025**
**MADRS**	1.14 (1.1–1.17)	**t(265.6) = 8.4**	**<0.001**	**0.024**	**0.031**
YMRS	1.02 (0.96–1.09)	t(242.8) = 0.6	0.525	0.081	0.088
Antidepressant	0.76 (0.44–1.29)	t(270.7) = −1	0.303	0.008	0.015
Anticonvulsant	1.27 (0.76–2.12)	t(269.2) = 0.9	0.362	0.013	0.02
Lithium Carbonate	0.98 (0.51–1.87)	t(271.1) = −0.1	0.94	0.007	0.014
Antipsychotic	0.8 (0.4–1.59)	t(264.7) = −0.6	0.523	0.027	0.034
**Anxiolytic**	2.12 (1.22–3.67)	**t(270.6) = 2.7**	**0.008**	**0.009**	**0.016**
**Any lifetime substance use disorder**	1.7 (1.02–2.83)	**t(259.4) = 2**	**0.043**	**0.041**	**0.049**
**BIS**	1.08 (1.06–1.1)	**t(268.4) = 6.7**	**<0.001**	**0.016**	**0.023**
**CTQ**	1.04 (1.02–1.05)	**t(261.2) = 4.7**	**<0.001**	**0.037**	**0.044**
**FAST**	1.08 (1.06–1.1)	**t(269.5) = 8.1**	**<0.001**	**0.012**	**0.019**
**MARS**	0.87 (0.78–0.96)	**t(234.2) = −2.7**	**0.008**	**0.099**	**0.107**
Type of WAIS	1.5 (0.78–2.89)	t(268.4) = 1.2	0.223	0.016	0.023

OR (95% CI): Odds Ratio (95% Confidence Interval); fmi: fraction of missing information,CGI: Clinical Global Impression scale, MADRS: Montgomery Åsberg Depression Rating Scale, YMRS: Young Mania Rating Scale, BIS: Barratt Impulsiveness Scale, CTQ: Childhood Trauma Questionnaire, FAST: Functioning Assessment Short Test, MARS: Medication Adherence Rating Scale, WAIS: Wechsler Adult Intelligence Scale
