# Supplementary material for: Exploring Clinical Correlates of Metacognition in Bipolar Disorders Using Moderation Analyses: The Role of Antipsychotics
Source: J Clin Med. 2021 Sep 24;10(19):4349. doi: 10.3390/jcm10194349 (PMC8509459; doi:10.3390/jcm10194349)
Supplement: Supplementary file 1 [file jcm-10-04349-s001.zip › Supplementary Table S5_revPR.pdf]

Supplementary Table S5. Results for the trivariable ordinal logistic regressions with cognitive complaints as the dependent variable and objective cognition, several successive clinical moderators, and the interaction between objective cognition and the clinical moderators as the independent variables. This table reports only the main effect of objective cognitive performance (the main effect of the clinical moderators and the interaction between objective cognition and the clinical moderators are reported in Supplementary Table A4 & Table 3 respectively).

Variable	OR (95% CI)	Statistic	p	λ	fmi
Age	1.45 (0.28–7.56)	t(255.7) = 0.4	0.661	0.051	0.058
Sex	0.69 (0.36–1.31)	t(254.9) = −1.1	0.255	0.053	0.06
Educational level	0.48 (0.03–7)	t(240.3) = −0.5	0.589	0.086	0.094
Diagnosis (Type 2/NOS vs Type 1)	0.74 (0.33–1.65)	t(239.4) = −0.7	0.46	0.088	0.096
Total number of mood episodes	0.87 (0.37–2.02)	t(108.9) = −0.3	0.735	0.393	0.404
**Predominant Polarity**	**0.31 (0.13–0.77)**	**t(155.8) = −2.5**	**0.012**	**0.265**	**0.274**
Age at onset	0.76 (0.19–3.09)	t(253.8) = −0.4	0.702	0.055	0.063
History of psychosis	0.63 (0.37–1.07)	t(227.5) = −1.7	0.086	0.114	0.121
Rapid cycling	0.67 (0.42–1.07)	t(257.3) = −1.7	0.095	0.047	0.054
CGI Severity	0.09 (0.01–1.41)	t(241.2) = −1.7	0.086	0.084	0.092
MADRS	0.72 (0.36–1.44)	t(254) = −0.9	0.356	0.055	0.062
YMRS	0.67 (0.38–1.19)	t(244.4) = −1.4	0.168	0.077	0.085
Antidepressant	0.74 (0.45–1.22)	t(261.7) = −1.2	0.237	0.035	0.042
Anticonvulsant	0.68 (0.42–1.11)	t(261.4) = −1.6	0.121	0.036	0.043
Lithium Carbonate	0.79 (0.48–1.29)	t(252) = −1	0.337	0.06	0.067
**Antipsychotic**	**0.48 (0.29–0.79)**	**t(257.8) = −2.9**	**0.004**	**0.045**	**0.053**
Anxiolytic	0.84 (0.51–1.39)	t(250.9) = −0.7	0.489	0.062	0.07
Any lifetime substance use disorder	0.65 (0.38–1.09)	t(251.5) = −1.6	0.103	0.061	0.068
BIS	0.13 (0.01–3.12)	t(248.4) = −1.3	0.21	0.068	0.076
CTQ	0.93 (0.2–4.21)	t(235.5) = −0.1	0.921	0.097	0.104
**FAST**	**0.44 (0.2–0.98)**	**t(259.7) = −2**	**0.044**	**0.04**	**0.048**
MARS	0.35 (0.08–1.48)	t(245.6) = −1.4	0.152	0.075	0.082
Type of WAIS	0.73 (0.2–2.67)	t(184.5) = −0.5	0.631	0.202	0.21

OR (95% CI): Odds Ratio (95% Confidence Interval); fmi: fraction of missing information, NOS: not otherwise specified; CGI: Clinical Global Impression scale, MADRS: Montgomery Åsberg Depression Rating Scale, YMRS: Young Mania Rating Scale, BIS: Barratt Impulsiveness Scale, CTQ: Childhood Trauma Questionnaire, FAST: Functioning Assessment Short Test, MARS: Medication Adherence Rating Scale, WAIS: Wechsler Adult Intelligence Scale
