# Supplementary figures and images for: Exploring Clinical Correlates of Metacognition in Bipolar Disorders Using Moderation Analyses: The Role of Antipsychotics
Source: J Clin Med. 2021 Sep 24;10(19):4349. doi: 10.3390/jcm10194349 (PMC8509459; doi:10.3390/jcm10194349)

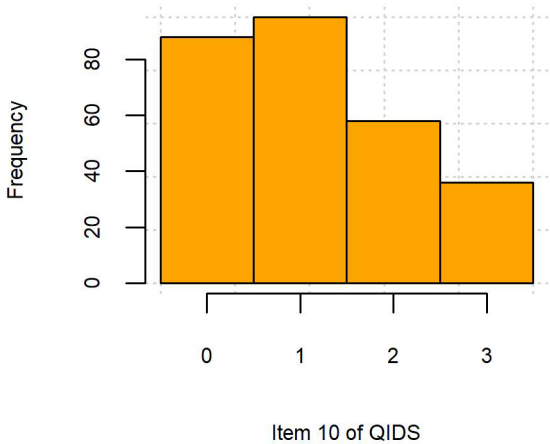

Supplement: Supplementary file 1 [file jcm-10-04349-s001.zip › Supplementary Figure S2_revPR.pdf]
